# Relationship of Structure and Function of DNA-Binding Domain in Vitamin D Receptor

**DOI:** 10.3390/molecules200712389

**Published:** 2015-07-07

**Authors:** Lin-Yan Wan, Yan-Qiong Zhang, Meng-Di Chen, Chang-Bai Liu, Jiang-Feng Wu

**Affiliations:** 1 Medical College, China Three Gorges University, 8 Daxue Road, Xiling District, Yichang 443002, China; E-Mails: wanlinyan0224@163.com (L.-Y.W.); zhangyanqiong@ctgu.edu.cn (Y.-Q.Z.); 15572753727@163.com (M.-D.C.); 2 Department of Pathogenic Biology and Immunology, Medical College, China Three Gorges University, 8 Daxue Road, Xiling District, Yichang 443002, China; 3 Hubei Key Laboratory of Tumor Microenvironment and Immunotherapy, China Three Gorges University, 8 Daxue Road, Xiling District, Yichang 443002, China

**Keywords:** VDR DBD, zinc finger structure, CTE, VDRE, nVDRE

## Abstract

While the structure of the DNA-binding domain (DBD) of the vitamin D receptor (VDR) has been determined in great detail, the roles of its domains and how to bind the motif of its target genes are still under debate. The VDR DBD consists of two zinc finger modules and a C-terminal extension (CTE), at the end of the C-terminal of each structure presenting α-helix. For the first zinc finger structure, N37 and S-box take part in forming a dimer with 9-*cis* retinoid X receptor (RXR), while V26, R50, P-box and S-box participate in binding with VDR response elements (VDRE). For the second zinc finger structure, P61, F62 and H75 are essential in the structure of the VDR homodimer with the residues N37, E92 and F93 of the downstream of partner VDR, which form the inter-DBD interface. T-box of the CTE, especially the F93 and I94, plays a critical role in heterodimerization and heterodimers–VDRE binding. Six essential residues (R102, K103, M106, I107, K109, and R110) of the CTE α-helix of VDR construct one interaction face, which packs against the DBD core of the adjacent symmetry mate. In 1,25(OH)_2_D_3_-activated signaling, the VDR-RXR heterodimer may bind to DR3-type VDRE and ER9-type VDREs of its target gene directly resulting in transactivation and also bind to DR3-liked nVDRE of its target gene directly resulting in transrepression. Except for this, 1α,25(OH)_2_D_3_ ligand VDR-RXR may bind to 1αnVDRE indirectly through VDIR, resulting in transrepression of the target gene. Upon binding of 1α,25(OH)_2_D_3_, VDR can transactivate and transrepress its target genes depending on the DNA motif that DBD binds.

## 1. Introduction

The vitamin D receptor (VDR), as a member of the steroid and nuclear hormone receptor (NR) superfamily, plays a vital role in regulating the metabolism of calcium and phosphorus and also implicates in regulating cellular proliferation, differentiation, apoptosis and adaptive/innate immune responses. Acting as a ligand-induced transcription factor, it can form homodimer or heterodimer with 9-*cis* retinoid X receptor (RXR) to regulate the expression of target genes through binding to specific sequence in the promoter or enhancer regions of 1α,25(OH)_2_D_3_ target genes [1]. The VDR consists of several functional domains. The highly variable N-terminal A/B domain is responsible for ligand-inducible VDR transactivated functions, but its structural elements are poorly defined. The central highly conserved core DNA-binding domain (DBD) and the less-conserved ligand-binding domain (LBD) at the C-terminal end are present in all members of the NR superfamily. Another flexible region connecting the DBD and the LBD is called the hinge domain [2]. Even though the VDR is divided into several functional domains, it plays a role as a whole.

As the most vital part of the VDR, the DBD is of central importance and sufficient for target gene discrimination and DNA binding. How every functional area plays a role and interacts with each other, or with VDR response elements (VDRE), is not yet clear. In this review, we will summarize the structure and the important functions of every functional area of the VDR DBD, and the interactions between VDR DBD and VDRE.

## 2. The Structure of the VDR DBD

**Figure 1:**
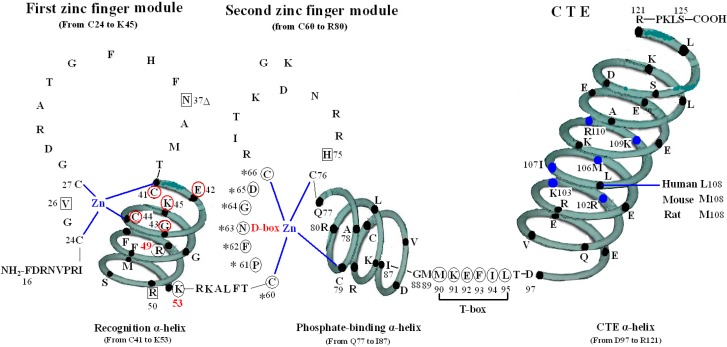
Structure of DNA-binding domain of the vitamin D receptor (VDR DBD) of human, mouse and rat. The amino acid sequences of VDR DBD of human, mouse and rat extend from residues 16 to 125 and are almost exactly the same except for L108 of human and M108 of rat and mouse. The VDR DBD consists of two zinc finger structures and a C-terminal extension (CTE). Three green three-dimensional spiral patterns show three α-helices in the VDR DBD. The solid dots within α-helices represent corresponding amino acids. Four boxes: P-box (from C41 to K45); S-box (R49 and K53); D-box (*) (from C60 to C66); and T-box (from M90 to L95). Three parts of amino acids in VDR DBD take part in forming dimerization interface for the interaction with the DBD of 9-*cis* retinoid X receptor (RXR). N37 and S-box (R49 and K53); T-box (from M90 to L95); and six solid blue dots in the CTE α-helix represent six amino acids which construct one interaction face packing against the DBD core of the adjacent symmetry mate (residues 34, 37, 90, 92 and 93).

The VDR DBD (residues 16–125) is made up of 66 highly conserved amino acid residues (from C24 to M89) and 44 relatively variable residues (from F16 to I23 and M90 to S125). It is almost exactly the same for the VDR DBD of human, rat and mouse, except for L108 of human beings and M108 of rats and mice. As the most conserved domain among the NR, the DBD consists of two zinc finger modules that form the core structure and a C-terminal extension (CTE), which either stabilizes DNA-binding by making contact with the minor groove of the DNA or participates in protein-protein contact of the DBD-dimer. There are three α-helices in the VDR DBD: recognition α-helix (residue C41 to K53), phosphate-binding α-helix (residue Q77 to I87) and CTE α-helix (residue D97 to R121). Within the two zinc finger modules, there are two structural zinc ions that are coordinated by four cysteines and buttress two perpendicular α-helices that pack together in the domain via their hydrophobic faces. The recognition α-helix is positioned for insertion into the major groove of the DNA. Side chains of the recognition α-helix contact the bases of the DNA’s major groove and are responsible for sequence specificity, while the second zinc finger module allows the VDR DBD to hetero- or homodimerize. Within the CTE, there exists a short six residue region referred to as the T-box, which has been confirmed to form dimerization interface for the interaction with the RXR DBD. Furthermore, the DBD dimerization has to be in sync with the formation of the hormone response element and the variations in spacer length and hexamer orientations of the response elements have to be accommodated by the second zinc finger and the CTE [3,4] (Figure 1).

## 3. The First Zinc Finger Structure

The first zinc finger structure is made up of 22 amino acid residues (from C24 to K45), within which exist a short five residues domain (from C41 to K45) named P-box [5,6,7]. P-box and its adjacent eight amino acids (from G46 to K53) construct a recognition α-helix. Furthermore, R49 and K53 in VDR are referred to as the DNA specific element, namely S-box [5].

**Figure 2:**
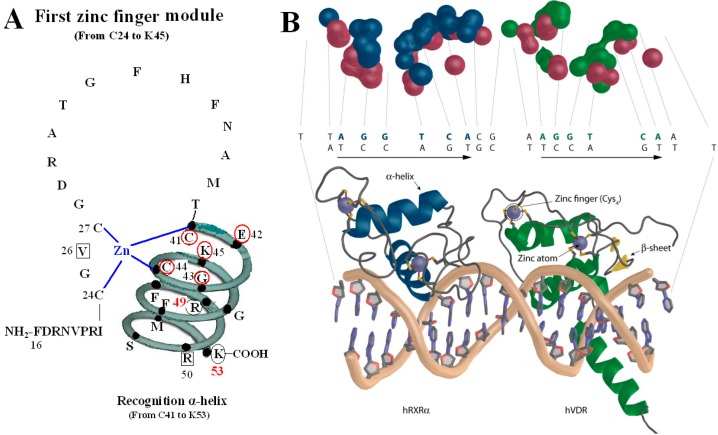
VDR DBD binds to VDR response elements (VDRE). (**A**) Amino acids of V26, P-box (from C41 to K45), R50 and S-box (R49 and K53) participate in binding to VDRE. (**B**) Pattern chart of the DBD complex of RXR-VDR on canonical DR3 element. (1) Bottom: The two zinc atoms are shown on spheres in light blue, RXR in blue and VDR in green. The α-helices are in gray and β-sheets in yellow. (2) Top: The contact atoms interacting between heterodimer and DNA are shown. DNA is shown in red, RXR in blue and VDR in green. Figure 2B adapted from Ferdinand Molnár and as originally published in Frontiers Media S.A. (2014) [9].

All the amino acids above are related to two kinds of function: on one hand, N37 and S-box take part in forming dimer with RXR (Figure 1) [8], on the other hand, V26, R50, P-box and S-box participate in binding with VDRE (Figure 2) [9,10]. N37 in VDR can form VDR-RXR heterodimer with R48, Q49, and R52 of RXR through H-bonds [11], while S-box plays a vital role in both dimerization and VDRE binding. For the S-box, neither R49 nor K53 is completely conserved throughout the NRs, but their roles may be receptor specific, or at least group specific. In fact, the position corresponding to R49 in VDR is occupied by either an arginine or its conservative replacement, lysine, in the NRs that bind DNA. S-box possesses specific binding of the VDR-RXR heterodimer to the natural VDRE, one of which R49 in S-box constitutes a critical determinant of VDRE recognition that operates in conjunction with the invariant base contact residues, K45 and R50. R49 and R50 are the only two residues that make both phosphate and base contacts [5]. Another residue in S-box, K53, is significant in VDRE binding, likely forming contacts with the phosphate backbone at the second and/or third position of the 3′ DNA half-element. The VDR mutant (E42A) lacks DNA binding ability for DR3-type-positive VDRE [12].

The P-box of the VDR DBD appears dispensable for binding to VDREs with an A, rather than a T, in the fourth position of the 3′ half-sites when it is replaced with a GR-like P-box that is GSV (gly-ser-val), which possesses a compensatory valine in the third position. It is likely significant that the S-box (R49 and K53) is a part of the VDR DNA-recognition α-helix, which also contains the P-box. Except for the structure as a whole, they also interact with each other in function. R49 and K53 are located in the same side of the recognition α-helix and act as a pair of residues that are required to complement the P-box in VDRE recognition. Sequence-specific interactions between VDR and the hexameric half-site are related to side chains of the recognition α-helix (residues 41 to 53) [5].

The function of V26 is to bind to VDRE because mutation of V26M in the VDR DBD exhibits normal ligand-induced binding to RXR and to coactivator, however, abolishes 1,25(OH)_2_D_3_-mediated transactivation, which results in the V26M mutation inhibited VDR binding to a consensus VDRE [13].

## 4. The Second Zinc Finger Structure

**Figure 3:**
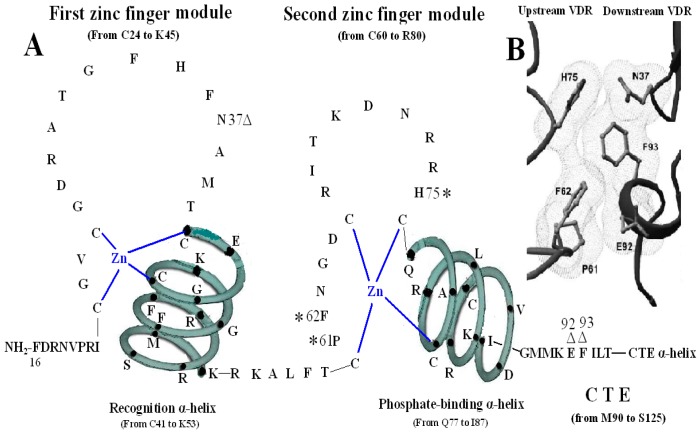
Homodimerization interface of VDR DBD. (**A**) The amino acids forming VDR homodimerization interface. 3 asterisks of H75, P61 and F62 form upstream dimmer interface and three triangles of N37, E92 and F93 form downstream dimmer interface. (**B**) Stereo view of the VDR homodimerization interface in a van der Waals surface representation. Figure 3B adapted from Shaffer and Gewirth (2002).

The second zinc finger structure consists of 21 amino acid residues (from C60 to R80) and four residues (from Q77 to R80) at the end of C-terminal end and its adjacent seven amino acids (from L81 to I87) construct the second α-helix of the VDR DBD. It has been confirmed that residue P61, F62 and H75 are vital in the structure of the VDR homodimer. The three residues of the upstream VDR and residues N37, E92 and F93 of the downstream of partner VDR form the inter-DBD interface. The six residues are nearly invariant among VDRs of all species, and the homodimer interface combined by the six residues is also unique among NRs. The mechanisms of association across the interfacial gap are via hydrogen bond between H75 and N37 and van der Waals contacts that produce a smooth, cater interface. P61 restricts the conformation of F62 on the upstream subunit and F93 pushes N37 to make a hydrogen bond with H75 of the opposite subunit. VDR DBD homodimer is relatively stable compared with the heterodimer of RXR-VDR DBD because the interfaces formed by the six residues are tightly packed and lack direct DNA support. Mutation of F62 and H75 may abolish cooperative assembly on DR3 elements [14,15,16,17]. However, a VDR-point mutant (P61T) failed to transrepress through 1αnVDRE but still retained ligand-induced transactivation through the positive VDRE (DR3-type VDRE). The mutant where proline is replaced with threonine at position 62 in the VDR DBD was also unable to interact with VDR interacting repressor (VDIR) *in vitro* [18]. The triple mutant of VDR (Pro61Ala, Phe62Ala, and His75Ala) does not impair heterodimer formation and DNA binding in the context of the full-length receptors [3,19,20] (Figure 3).

The D-box, encompassing the N-terminal base just between cys60 and cys66 of the second zinc finger structure, has a weaker dimerization function than the P-box and it is possibly critical for ligand-induced interaction and presumably the transrepression of VDIR [21].

## 5. The CTE of the VDR DBD

The CTE, which consists of 36 amino acid residues (from M90 to S125), is the extension of VDR DBD and at the beginning of the CTE locates a short six residue region (from the M90 to L95), called the T-box, which has been confirmed to form a dimerization interface for the interaction with the RXR DBD (Figure 1) [22]. Adjacent to the T-box is a long CTE α-helix, which is made up of 25 residues (from D97 to R121) [23].

It has been demonstrated that the T-box of the CTE played a critical role in heterodimerization and heterodimers–VDRE binding. Furthermore, T-box has also been verified essential for directing the orientation of the long α-helical part of the hinge region. Among the six residues of the T-box, K91 and E92 form salt bridges with D39 and R38, respectively, in RXR, while F93 and I94 takes part in all the functions of the CTE above [24,25].

Functional analysis about T-box of VDR has clarified that F93 is critical on both DR3-type and ER9-type VDRE and F93 is unique for VDR in comparison with the other NR superfamily members. Under physiological conditions, I94 is revealed to be vital on both VDRE types and the influence of I94 on protein–DNA complex formation is found to rely on ionic strength of the binding reaction and the nature of the VDRE. Except for dimerization and VDRE binding, both F93 and I94 have been recognized compactly related to the decision of the α-helix orientation. Taken together, F93 and I94 are not only the most vital residues of the T-box, but may also be critical in the context of the whole hinge region, as they not only have a role in the dimerization interface of the DBDs, but also appear to direct the relative orientation of the DBD to the LBD [24].

During the VDR-RXR heterodimer formation, six important residues (R102, K103, M106, I107, K109, and R110) of the CTE α-helix of VDR construct one interaction face, which packs against the DBD core of the adjacent symmetry mate (residues 34, 37, 90, 92 and 93) and is stabilized primarily by non-specific Van Der Waal’s contacts (Figure 1) [4]. Moreover, biochemical studies have implicated the basic residues from 102 to 104 and from 109 to 111 in the CTE α-helix of the VDR DBD as essential for both DNA binding and transactivation and they also increases the affinity by aiding in nonspecific interaction with the DNA backbone and minor groove [20].

Taken together, this revealed the necessary VDR residues in the S-box and CTE, respectively, and in combination with VDR P-box amino acids, confer the VDR-RXR with unique DNA binding properties [5].

## 6. Vitamin D Response Elements

As a ligand-activated transcription factor, VDR can bind to specific sequence in the promoter of vitamin D target genes, commonly referred to vitamin D response elements. Vitamin D response elements can be divided into two groups, one is positive vitamin D response elements (VDREs), which contain DR3-type VDRE and ER9-type VDRE; the other is negative vitamin D response elements (nVDREs), which also consist of DR3-like nVDRE and 1αnVDRE [18].

VDREs are typically formed by a tandem repeat of two hexameric core binding half-sides arranged either as direct repeats spaced by 3nt (DR3) or everted repeat with nine spacing nucleotides (ER9). NRs recognized generic hexad DNA half-sites typically consisting of two primary categories, 5′-AGGTCA-3′ and 5′-AGAACA-3′, in which the direct repeats differ and facilitate selective responsive element binding by the receptors. DR3-type VDRE contains directly repeated 5′-AGGTCA-3′ motifs separated by 3nt [26,27].

In the absence of DNA target, the DBDs themselves do not form dimerization, but ligand binding strengthens dimer formation [2]. Unliganded VDR can bind to the response element as a homodimer, while upon binding of the ligand, VDR forms a heterodimer with RXR to play role as a whole. On DR3-type VDRE, VDR DBD is easy to form homodimers with itself and is not easy to form heterodimers with RXR DBD [3,20]. Moreover, on DR3-type VDRE, the RXR DBD is positioned on the 5′-side of the DNA, while the VDR DBD is on the 3′-side and both VDR DBD and RXR DBD are located roughly on the same side of the DNA. Contrary to this, VDR-RXR heterodimers appear to be more conveniently formed on ER9-type VDRE than on DR3-type VDRE and the DBDs are nearly on opposite sites of ER9-type VDREs [4].

In 1,25(OH)_2_D_3_-activated signaling, the VDR-RXR heterodimer may also mediate target gene, such as parathyroid hormone-related peptide (PTHRP) [28], down-regulatory by binding directly to a 16-bp sequences (GGGTGGAGAGGGGTGA), which consists of an almost perfect repeat separated by a 3-bp “spacer” [29]. One of repeat sequences (GGGTGA) is entirely homologous to a half-site of the VDRE, and another non-consensus direct repeat (GGGTGGA) is located in 3-bp upstream of this motif, therefore, it is called DR3-liked nVDRE [30,31].

1αnVDRE is another novel nVDRE which has been identified in the *CYP27B1* promoter [32] and *CYP21A2* promoter [33]. The interaction of liganded VDR-RXR heterodimer with the 1αnVDRE is an indirect binding, which occurs via another bHLH-type transcription factor, the designated VDIR [32]. In the absence of 1α,25(OH)_2_D_3_, the Williams syndrome transcription factor (WSTF), VDR and VDIR interact with 1αnVDRE in the promoter for transactivation, which are potentiated by recruiting p300/CBP and histone acetylase (HAT) co-activator, inducing the serine residues of the transactivation domain phosphorylation via PKA signaling pathway [33]. While upon binding of 1α,25(OH)_2_D_3_ to VDR, the VD-VDR/RXR-VDIR-1αnVDRE complexes cause the dissociation of p300/CBP-HAT coactivator and recruitment of histone deacetylase (HDAC) co-repressor complex components, resulting in ligand-induced transrepression [18].

## 7. Conclusion and Perspectives

The VDR DBD consists of two zinc finger modules and a CTE, at the end of C-terminal of each structure presents one α-helix. For the first zinc finger structure, on the one hand, N37 and the S-box take part in forming dimer with RXR and on the other hand, V26, R50, the P-box and the S-box participate in binding with VDRE. For the second zinc finger structure, P61, F62 and H75 are essential in the structure of the VDR homodimer with residues N37, E92 and F93 of the downstream of partner VDR, which form the inter-DBD interface. It is possibly critical for ligand-induced interaction and presumably the transrepression of VDIR. The T-box of the CTE plays a critical role in heterodimerization and heterodimers–VDRE binding. F93 and I94 are not only the most vital residues of the T-box, but may also be critical in the context of the whole hinge region, as they do not only have a role in the dimerization interface of the DBDs, but also appear to direct the relative orientation of the DBD to the LBD. During the VDR-RXR heterodimer formation, six important residues (R102, K103, M106, I107, K109, and R110) of the CTE α-helix of VDR construct one interaction face, which packs against the DBD core of the adjacent symmetry mate (residues 34, 37, 90, 92 and 93) and is stabilized primarily by non-specific Van Der Waals contacts [4].

Unliganded VDR can bind to the response element as a homodimer, while upon binding of the ligand, VDR forms a heterodimer with RXR to play role as a whole. In 1,25(OH)_2_D_3_-activated signaling, the VDR-RXR heterodimer may bind to DR3-type VDRE and ER9-type VDREs of its target gene directly resulting in transactivation and also bind to DR3-liked nVDRE of its target gene directly resulting in transrepression. Except for this, 1α,25(OH)_2_D_3_ ligand VDR-RXR may bind to 1αnVDRE indirectly through VDIR, resulting in transrepression of target gene. Upon binding of 1α,25(OH)_2_D_3_, VDR can transactivate and transrepress its target genes depending on the DNA motif it is binding.

There are 14,548 sites bound by VDR through bioinformatic analysis of the extent of cistrome intersection and 16%–21% of these putative binding sites are found at gene promoters [34]. Certainly, it needs to be confirmed by experiment whether these sites can be bound effectively and whether their corresponding genes are target genes. It is worth noting that some target genes of VDR have many binding sites in their promoter, for example, in the promoter of *CYP27B1* gene, there are 13 VDRE and one nVDRE, which present a crosstalk of distal and proximal promoter regions during the transcriptional regulation [32]. Therefore, it is the wide regulating range of VDR that will cause many side effects when using 1α,25(OH)_2_D_3_ to treat diseases. It will be a feasible strategy to mutate the essential amino acids of VDR DBD to decrease its ability to bind some kinds of VDRE or nVDRE.
